# Dynamics of voluntary attention indicators of adolescents with endogenous mental pathology during treatment

**DOI:** 10.1192/j.eurpsy.2021.594

**Published:** 2021-08-13

**Authors:** A. Koval-Zaytsev

**Affiliations:** Department Of Child Psychiatry, Federal State Budgetary Scientific Institution “Mental Health Research Center”, Moscow, Russian Federation

**Keywords:** adolescents, voluntary attention, endogenous mental pathology, dynamic research

## Abstract

**Introduction:**

The study of voluntary attention is necessary to assess the effectiveness of therapeutic, psychotherapeutic and psychocorrective measures. Evaluation of the effectiveness of treatment by analyzing the dynamics of indicators of voluntary attention of adolescents with endogenous mental pathology is necessary for the development of personalized patient management

**Objectives:**

To analyze the dynamics of indicators of voluntary attention to memory in adolescents with endogenous mental pathology during treatment.

**Methods:**

clinical-catamnestic, pathopsychological methods. We examined 153 patients aged 12-16 years (average-13.7 years) with diagnoses of F21.3, F21.4, F20.8xx3 (ICD-10). The comparison group consisted of 143 healthy peers. Methods children’s color train test, Schulte tables. All subjects were examined twice – at the beginning of therapy and at discharge from the clinic.

**Results:**

Analysis of the results of comparing the dynamics of attention in adolescents with endogenous mental diseases during the initial examination and during repeated examination showed that adolescents with diagnoses of F21.3, F21.4, as well as with a diagnosis of F20.8xx3 improve their attention indicators during repeated examination (at p< 0.01). Adolescents of the experimental group showed better results compared to the control group, which indicates that there is a positive therapeutic dynamics of attention in sick adolescents. Comparison of therapeutic dynamics of attention of adolescents with endogenous mental diseases depending on the diagnosis revealed significant differences. Adolescents from the F21 group performed better than the F20 group (at P< 0.01).

**Conclusions:**

The Study showed the effectiveness of the choice of this methodological tool in assessing the therapeutic dynamics of patients.

